# Poly(A) RNAs Including Coding Proteins RNAs Occur in Plant Cajal Bodies

**DOI:** 10.1371/journal.pone.0111780

**Published:** 2014-11-04

**Authors:** Janusz Niedojadło, Ewa Kubicka, Beata Kalich, Dariusz J. Smoliński

**Affiliations:** Department of Cell Biology, Faculty of Biology and Environment Protection, Nicolaus Copernicus University, Torun, Poland; University of Toronto, Canada

## Abstract

The localisation of poly(A) RNA in plant cells containing either reticular (*Allium cepa*) or chromocentric (*Lupinus luteus*, *Arabidopsis thaliana*) nuclei was studied through *in situ* hybridisation. In both types of nuclei, the amount of poly(A) RNA was much greater in the nucleus than in the cytoplasm. In the nuclei, poly(A) RNA was present in structures resembling nuclear bodies. The molecular composition as well as the characteristic ultrastructure of the bodies containing poly(A) RNA demonstrated that they were Cajal bodies. We showed that some poly(A) RNAs in Cajal bodies code for proteins. However, examination of the localisation of active RNA polymerase II and *in situ* run-on transcription assays both demonstrated that CBs are not sites of transcription and that BrU-containing RNA accumulates in these structures long after synthesis. In addition, it was demonstrated that accumulation of poly(A) RNA occurs in the nuclei and CBs of hypoxia-treated cells. Our findings indicated that CBs may be involved in the later stages of poly(A) RNA metabolism, playing a role storage or retention.

## Introduction

Cajal bodies (CBs) are multifunctional domains present within the nuclei of plant and animal cells. CBs are approximately 0.5–1.0 µm in diameter, but their size and number depend on the cell type, cell cycle, and metabolic activity [1]. CBs have been shown to be involved in RNA-related metabolic processes such as snRNP and snoRNP biogenesis, maturation, and recycling, histone mRNA processing and telomere maintenance [2], [3]. However, the list of processes with which CBs have been associated is dynamically expanding. It has recently been shown that CBs are engaged in such processes as siRNA biogenesis [4], deadenylation of snoRNA [5], and the pathophysiology of fragile X syndrome [6].

CBs are associated with particular stages of snRNA biogenesis, such as transcription, those involving loud export factors, modifications and snRNP assembly [7]. U1 and U2 genes can often be found in close proximity to CBs. The association of CBs with U2 genomic loci has been shown to be dependent on active transcription and to be mediated by nascent pre-U2 RNA [8]. SnRNA precursors micro-injected into the nuclei of *Xenopus laevis* oocytes temporarily concentrate in CBs [9]. SnRNAs may enter CBs for proper loading of the export factors PHAX and CRM1, which then transport snRNAs to the cytoplasm. After transport to the cytoplasm, snRNAs undergo 5′-cap hypermethylation, and acquired Sm proteins are reimported into nuclei and into CBs to complete their maturation [2]. This process includes the assembly of snRNP-specific proteins and snRNA methylation and pseudouridylation by scaRNPs [10], [11], in addition to the formation of a complex consisting of U4, U5, and U6 snRNPs referred to as tri-snRNP by SART3/p110 proteins [12], [7].

The coilin protein is the key component and marker of Cajal bodies. The homolog of coilin in plants, Atcoilin shows a strong affinity for snRNA, but also for non-specific RNA [13]. It has been demonstrated that the interaction between coilin and U1 snRNA leads to multimerisation of these proteins, which is a process induced by structural remodelling of the N-terminal portion of the molecule [13]. Symmetrical dimethylation of arginines and the phosphorylation status of coilin are essential for the formation and composition of CBs [3]. The symmetric dimethylarginine (sDMA) modification strongly increases the affinity of coilin of SMN (survival motor neuron) for RG dipeptide repeats [14], [15]. Coilin hypomethylation results in the delocalisation of SMN to twin structures referred to as the gemini of CBs, or gems [16], [17]. Coilin phosphorylation plays a crucial role in recruiting U snRNPs to CBs. In the model of Toyota et al. [18], snRNPs are imported into the nucleus in association with the SMN complex. In the nucleus, hypophosphorylated coilin recruits the SMN complex to the CB, and hyperphosphorylation then allows the transfer of snRNPs from SMN to coilin, where they can be used by the modification machinery in the CB.

Although CBs are involved in many basic processes such as snRNA maturation, they have not been observed in all types of eukaryotic cells. Cajal bodies are absent from some adult cell types, including smooth and cardiac muscle, endothelial cells, dermal and epidermal cells, despite the presence of SMN and coilin in these tissues [19]. However, gems and CBs are present in all human foetal tissues, even those that lack gems/CBs in their adult form. Tucker et al. [20] showed that coilin-homozygous mice are both viable and fertile, but the numbers of mice obtained are significantly reduced when crossed with inbred backgrounds. Similarly, in coilin-homozygous *Arabidopsis thaliana* mutants, the growth phenotype is not observed in mutants lacking observable CBs [21]. These results suggest that coilin and Cajal bodies are not essential for the functioning of a cell. However, Strzelecka et al. [22] demonstrated a significant contribution of CBs in the embryonic development of zebrafish. In this model, disruption of morphologically defined CBs via coilin depletion leads to developmental arrest. In coilin-depleted embryos, there is a ∼50% reduction in the levels of snRNPs produced *de novo*. In addition to the maturation of individual snRNAs, di- and tri-snRNP assembly also occurs in CBs. An *in vivo* kinetics analysis revealed the production of 3.8 tri-snRNPs per second, which is approximately 10-fold faster than in the surrounding nucleoplasm [23]. Therefore, CBs have been proposed as a site that speeds up snRNP assembly by concentrating necessary components [7], which may make a crucial contribution to foetal tissues or embryonic and intensively dividing cells.

In a previous study, Kołowerzo et al. [24] observed that polyadenylated RNA localises to CBs in diplotene microsporocytes in larch (*Larix deciduas* Mill). In the present study, our purpose was to clarify whether this phenomenon occurs only in the generative line, or if it is also commonly observed in somatic cells. We localised poly(A) RNAs in reticular and chromocentric types of nuclei in the roots of plants via *in situ* hybridisation, and we evaluated whether these transcripts were protein coding and whether CBs may serve as a site for their synthesis. Measurements of the amount of poly(A) RNA were conducted in root cell nuclei under natural and stress conditions.

## Materials and Methods

Bulbs of *Allium cepa* L. (Horticulture Farm in Torun; Poland) were placed on a wire mesh covering a container full of tap water so that only the root blastema was exposed to water. Two or three days after the culture was initiated, the bulbs possessed roots 1–2 cm in length. *Lupinus luteus* cv Zeus (Torseed SA Torun; Poland) seeds were soaked in water for 5 h and subsequently germinated at 21°C for 3–4 days on water-soaked tissue paper.

Meristems of *Allium cepa* and *Lupinus luteus* roots were excised under water and fixed in 4% paraformaldehyde in 50 mM Pipes buffer, pH 7.0, for 12 h at 4°C. The fixed roots were washed three times for 15 min in Pipes buffer and for 15 min in PBS buffer.

*Arabidopsis thaliana* Col-0 seeds were sterilised and sown in 2/3 Murashige and Skoog medium supplemented with 0.7% (w/v) agar. The growth conditions involved continuous light with an irradiance of 60 µmol m^−2^ s^−1^ at 22°C. Meristems of 14-day-old *Arabidopsis thaliana* roots were excised under water and fixed in the same manner as the *Allium* and *Lupinus* roots.

For double localisation assays, onion and lupine root tips were sectioned under water into 50 µm-thick sections using a Vibratome Leica VT1200. The sections were then placed in embryo dishes and treated with 2% cellulase (Onuzuka R-10) and 25 U/ml pectinase (Sigma) in 0.01 M citric buffer, pH 4.8, for 17 min for *Allium* and 25 min for *Lupinus* root cells at 35°C. Then, the sections were rinsed with PBS and treated with 0.1% Triton ×100 solution in PBS buffer, pH 7.2, for 10 min. To induce hypoxia, *Lupinus* seedlings were stressed by submersion in tap water for 3 h. After fixation, the roots were placed a citric acid-buffered digestion solution (pH 4.8) containing 9% cellulase (Onuzuka R-10) and 35 U/ml pectinase (Sigma) for 90 min. After rinsing with PBS and distilled water, the root tips were squashed onto slides.

### Double labelling with *in situ* hybridisation of poly(A)

After rinsing with PBS, TritonX100-treated sections and protoplasts were incubated with the primary antibody mouse anti-PANA (1∶100) [25] or rat serine 2 of RNA polymerase II (1∶100) (Chromotec; Germany) in 1% BSA in PBS overnight. The slides were then washed in PBS and incubated with the appropriate secondary antibodies diluted in 1% BSA in PBS buffer (a goat anti-mouse IgG antibody labelled with Alexa Fluor 488 (Molecular Probes, NY, USA) or a goat anti-rat IgG antibody labelled with Alexa Fluor 488 (Molecular Probes, NY, USA). Then, *in situ* hybridisation was performed. After a 1-h prehybridisation, hybridisation was conducted for at least 12 h at 26°C in hybridisation buffer (Sigma-Aldrich) with 30% formamide and antisense poly(A) DNA—5 Cy3 T(T)29 3 at a concentration of 50 pmol/ml. The slices were subsequently washed in 4× SSC, 2× SSC, and 1× SSC. For FISH double labelling (U2 snRNA-poly(A) RNA), the two probes were applied simultaneously in the hybridisation medium, and hybridisation was conducted at 30°C using an antisense DNA probe against U2 snRNA 5′ rhodamine green: ATATTAAACTGATAAGAACAGA TACTACACTTG.

After fixation and rinsing in Pipes and PBS, the roots of *Arabidopsis thaliana* U2B”:GFP and Atcoilin:mRFP plants were treated with TritonX100 (1∶1000 in PBS) for 14 min. Then, they were subjected to the same *in situ* hybridisation protocol applied for the sections, described above, using 5'-Cy3- or rhodamine green-labelled DNA probes for poly(A) RNA. DNA was stained with 4,6-diamidino-2-phenylindole (DAPI) (Sigma-Aldrich). The control reaction was performed in the same manner, but using the hybridisation buffer without probes, and the primary antibodies were omitted.

### Double labelling of Sm proteins and transcripts in lupine

After digestion and Triton ×100 treatment, the sections were incubated with anti-Sm antibodies (1∶100; AF-ANA human serum, a gift from A. Pombo), then washed in PBS and incubated with a Cy2-labelled mouse anti-human antibody (Sigma-Aldrich). Next, *in situ* hybridisation was performed with DNA probes labelled with Cy3 (GenoMed Poland) for pectin methylesterase (5′TTCTCTGTATGTACCAGCTTTTATGTG3′), a cytokinin-specific binding protein (5′GAGTATGTAGCTCTCCTATTGCAGATAAT3′) cyclin B1 (5′CAATATCCTTAAGTACCCTCCTATTTCTT3′) and peroxidase (5′ ATCTTTGTAGTAATTATTGTCGAAACGAT3′) at a concentration of 50 pmol/ml in hybridisation buffer (Sigma-Aldrich) with 30% formamide. In case simultaneously *in situ* hybridisation with four probes at individual concentrations of 25 pmol/ml. *In situ* hybridisation was performed at 40°C for at least 12 h. The sections were then washed in 4× SSC and 2× SSC, and DNA staining was performed. The control reaction was conducted in the same manner as for double labelling with *in situ* hybridisation of poly(A).

### *In situ* hybridization at electron microscopy

The vibratome sections of roots fixed in 4% paraformaldehyde in 50 mM Pipes buffer, pH 7.0, for 12 h at 4°C after treatment with 0.1% saponin in PBS, were subjected to hybridisation using an LNA probe against poly(A) RNA labelled with digoxygenin at the 5′ and 3′ ends. For hybridisation, the probes were resuspended in hybridisation buffer (Sigma-Aldrich) with 30% formamide at a concentration of 20 nM. Hybridisation was performed for 12 h at 45°C. The sections were washed in 4× SSC and 2× SSC and post-fixed in 4% paraformaldehyde in Pipes buffer for 1 h at 4°C. Then, the sections were dehydrated, infiltrated, embedded and sliced according to Niedojadło et al. [26]. The grids with sections were treated for 12 h at 4°C with an anti-digoxygenin antibody (Roche) diluted 1∶100 in 1% BSA in PBS. A colloidal gold-conjugated secondary antibody was incubated with the sections for 45 min at 35°C. Finally, the sections were stained with 2.5% uranyl acetate (20 min) and, for some samples, with 2.5% lead citrate and examined using TEM (Joel 1010) at 80 kV.

### Immunodetection of 5-bromouracil incorporated and poly(A) RNA

After 3–4 days of culture, lupine seedlings were transferred to Petri dishes with 5-bromouarcil in tap water (20 mM, Sigma-Aldrich) soaked tissue paper for 2, 4, 5 or 7 h. The roots were fixed and sectioned, and the sections were treated according to the above protocol. Then sections were incubated with anti-BrUTP antibodies (F. Hoffmann-LaRoche Ltd., Rotkreuz, Switzerland) (diluted 1∶1000) overnight at 4°C, then washed in PBS and incubated with goat anti-mouse antibodies labelled with Alexa 488 (Molecular Probes, NY, USA). Next, *in situ* hybridisation of poly(A) RNA was performed according to the protocol described for double localisation with poly(A) RNA. Control reactions were conducted without the primary antibody. DNA was stained with 4,6-diamidino-2-phenylindole (DAPI; Fluka).

### Quantitative evaluation of fluorescence signals

To calculate the fluorescence signal resulting from the *in situ* hybridisation of poly(A) RNA, 53 cells from three different experiments were analysed. Similar to all of the results presented in this report, these results were registered and analysed using a Nikon PCM-2000 confocal microscope and a fluorescence inverted Nikon Eclipse TE 2000-E microscope. The analysis was performed using NIS-Elements AR3.00. The obtained data were corrected for background autofluorescence, as determined based on the signal intensity in the negative control. The non-parametric rank-based Kruskal–Wallis test was used to compare multiple groups, and when significant differences were detected, the Mann–Whitney test was applied to compare the two experimental groups. The tests were performed using GraphPad Prism 5 software. P <_0.05 was considered significant.

For Pearson correlation analysis, rectangular regions of interest were drawn to cover a single CB and its surroundings (1–4 µm^2^), and images analysis were conducted using NIS software. Representative examples are shown in the results. For each experiment, the 10 Cajal bodies were analyzed. This analysis was repeated three times, and the average and standard error were compiled.

## Results

**Figure 1:**
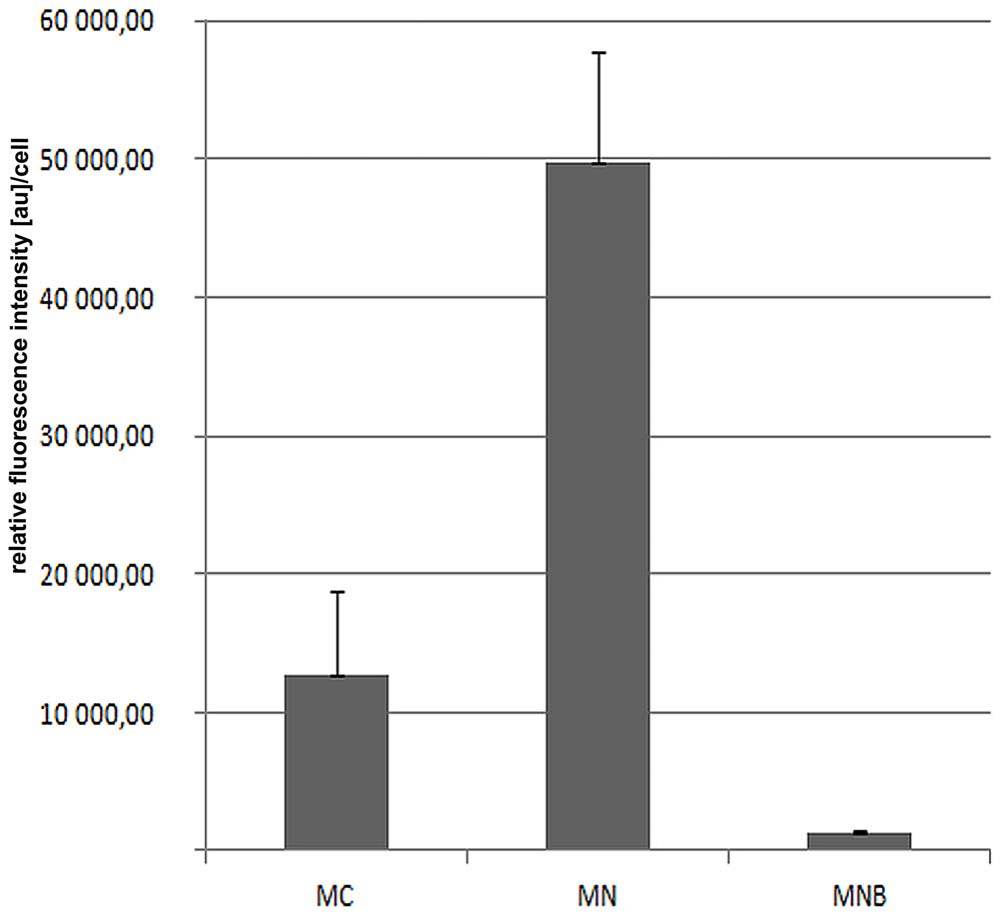
Analysis of the fluorescence intensity resulting from *in situ* hybridisation of poly(A) RNA in the meristematic cells of lupines roots. There were significant differences in intensity (p<0.05) between the cytoplasm (MC), nucleus (MN) and nuclear bodies (MNB).

**Figure 2:**
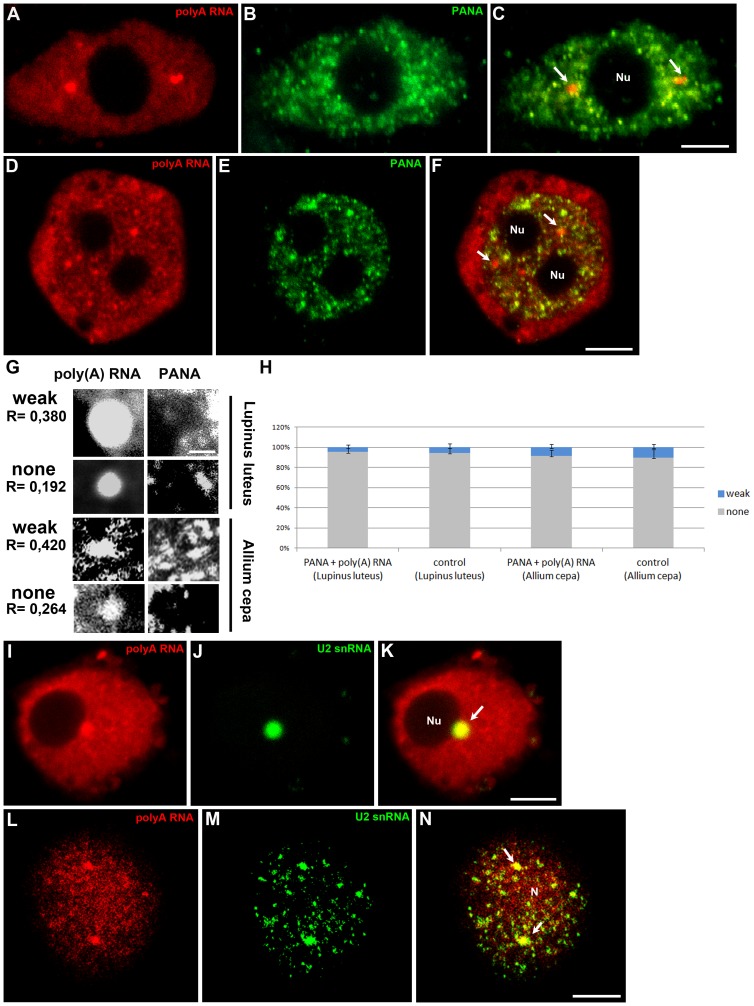
Double labelling of poly(A) RNA and the PANA antigen in the chromocentric (*Lupinus*) (A–C) and reticular (*Allium*) (D–F) nuclei of root cells. In the nucleoplasm of both species, poly(A) RNA is present in nuclear structures (arrows) and does not colocalise with speckles. Representative examples of Pearson correlation coefficients for weak and non-colocalisation of poly(A) mRNA with the PANA antigen in *Lupinus luteus* and *Allium cepa* cells (G). A scale bar representing 2 µm is shown. The percentages of weak and non-colocalisation of poly(A) RNA-rich bodies with the PANA antigen are indicated by the Pearson correlation coefficient (H). Error bars represent standard error. Double labelling of poly(A) RNA and U2 snRNA in *Lupinus* (I–K) and *Allium* (L–N) cells. Accumulation of poly(A) RNA in nuclear bodies rich in U2 snRNA (arrows). Bar, 5 µm. N-nucleus, Nu- nucleolus

We have shown that in the cells of plant roots, a considerably larger amount of poly(A) RNA is present in the nucleus than in the cytoplasm (up to more than 80% in lupine) (Figure 1). In the nucleoplasm, the signal forms clusters of different sizes, in both chromocentric (*Lupinus luteus*) (Figure 2A, C) and reticular (*Allium cepa*) nuclei (Figure 2D, F). Some of these clusters resemble nuclear bodies in terms of their shape and distribution. In lupine, the nuclear bodies enriched in poly(A) mRNA were more numerous and smaller in meristematic cells, whereas there were isolated but larger examples observed in differentiated cells of the roots (Figure S1A, B). These round clusters were observed in 87% of lupine root cells. Quantitative measurements have shown that in the meristematic cells of lupine plants, approximately 3.5% of the nuclear pool of the poly(A) RNA occurs in structures resembling nuclear bodies (Figure 1).

As many authors have demonstrated the presence of poly(A) RNA in splicing speckles in animal cells [27], we examined the colocalisation of poly(A) RNA with PANA antigens, a marker of speckles that correspond to interchromatin granules [25]. The results showed that the structures with the highest concentration of poly(A) RNA do not exhibit strong colocalisation with PANA antigens in the nuclei of either *Lupinus luteus* (Figure 2A–C) or *Allium cepa* (Figure 2D–F) roots. To evaluate the degree colocalisation, we assessed the Pearson correlation coefficient (R) between the poly(A) mRNA FISH stain and PANA immunofluorescence in individual structures rich in poly(A) mRNA. This analysis indicates the degree of poly(A) mRNA enrichment in each nuclear body compared with the surrounding regions. A weak correlation was considered to be indicated by 0,38>R>0,19 and 0,42>R>0,26 in *Lupinus luteus* and *Allium cepa*, respectively (Figure 2G). Strong colocalisation was not observed. This analysis confirmed that nuclear bodies containing poly(A) RNA do not colocalise with speckles (Figure 2H). As a control for random colocalisation, we also examined the correlation between the two investigated antigens overlaying a poly(A) RNA nuclear image on top of a PANA antigen immunofluorescence image, which was obtained from a different nucleus. Pearson correlation analysis also did not reveal any significant colocalisation in the control (Figure 2H).

**Figure 3:**
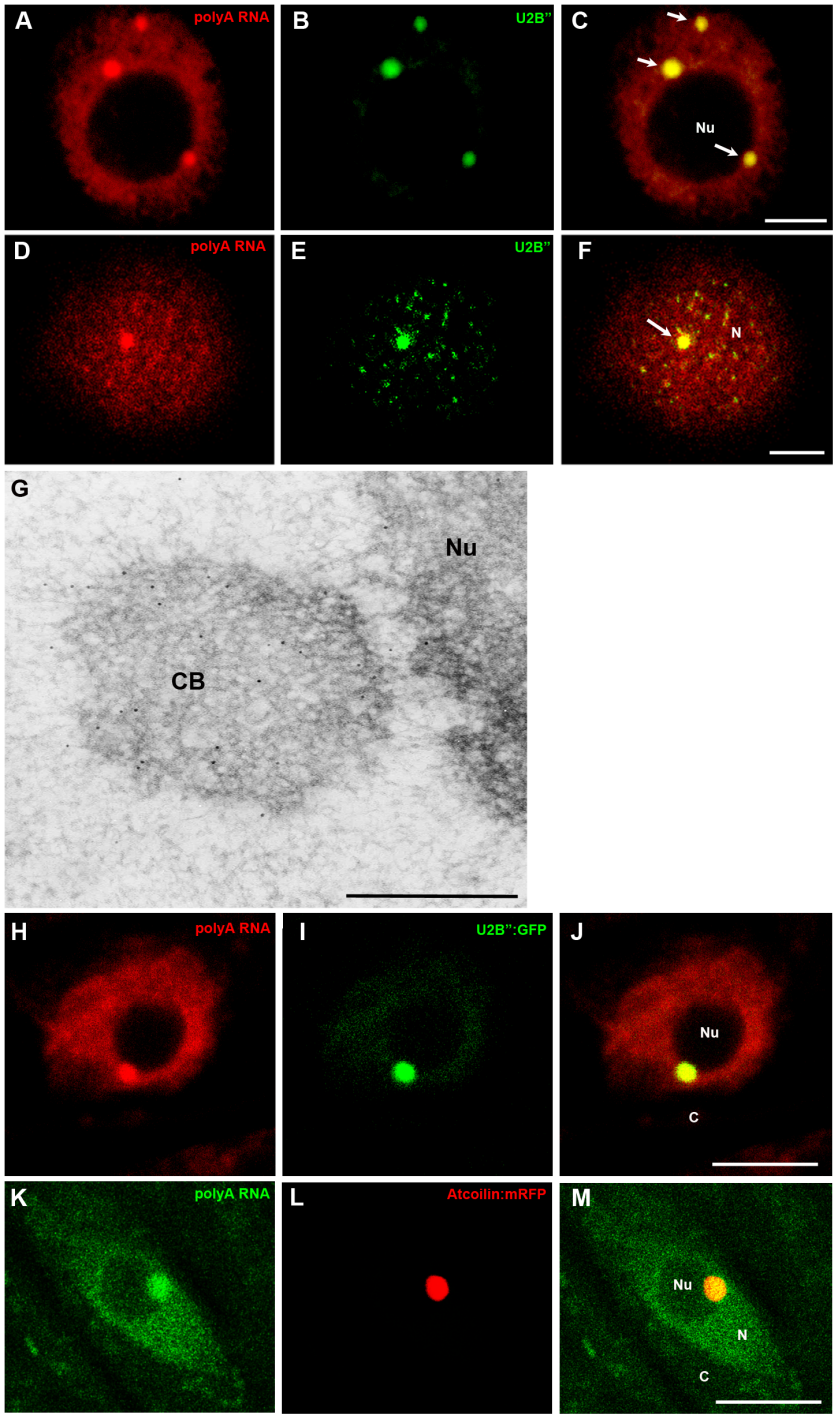
Double labelling of poly(A) RNA and the U2B” protein in *Lupinus* (A–C) and *Allium* (D–F) root cells. Poly(A) RNA present within Cajal bodies stained an anti-U2B” antibody (arrows). Immunogold labelling of poly(A) RNA in the Cajal bodies (CBs) of *Lupinus* root cells. Gold particles were mainly observed in the Cajal body (CB) (G). Simultaneous localisation of U2B”:GFP and poly(A) RNA in transgenic *Arabidopsis thaliana* root cells. Strong colocalisation in Cajal bodies (H–J). Localisation of poly(A) RNA with Atcoilin:RFP in *Arabidopsis thaliana* root cells. Accumulation of poly(A) in Cajal bodies (K–M). Bar, 5 µm. N-nucleus, Nu- nucleolus, C- cytoplasm.

Next, we tested whether the bodies rich in poly(A) mRNA colocalise with snRNA, which mainly occurs in the perichromatin region and Cajal bodies in plants [26]. The strongest colocalisation was observed in single round structures in *Lupinus luteus* upon double labelling of mRNA and U2snRNA via *in situ* hybridisation (Figure 2I–K). While U2snRNAs are concentrated in structures of different sizes in onion cells, only the largest, resembling nuclear bodies, colocalise with poly(A) RNA (Figure 2L–N). Similar results were obtained using antibodies against Sm proteins (Figure S1C–H). The round morphology, size, and average number per nucleus of the poly(A) mRNA and snRNP-containing structures are similar to Cajal bodies. As a homologous sequence to coilin, a marker of CBs, is only known in *Arabidopsis thaliana*, to identify these structures, antibodies against U2B” proteins were additionally employed. U2B” component, such as U2 snRNA and Sm proteins, are considered markers of CBs in plant cells [28], [29], [30]. Double labelling with anti-U2B” antibodies showed that the CBs present in lupine roots (Figure 3A–C) and onion (Figure 3D–F) cells contain poly(A) RNA. Additionally, the localisation of poly(A) RNA in the lupine cells was examined using electron microscopy. As it is usually difficult to achieve a strong signal, following the detection of poly(A) RNA in plant cells using an electron microscope, we conducted *in situ* hybridisation with oligo LNA probes. A strong poly(A) RNA signal was observed in Cajal bodies (Figure 3G). Despite the relatively low signal in the nucleus, some gold grains were observed in the nucleoplasm surrounding CBs. To confirm the presence of poly(A) RNA in Cajal bodies, *in situ* hybridisation in *Arabidopsis thaliana* U2B":GFP and Atcoilin:mRFP lines was performed. *In situ* hybridisations were conducted using fixed roots, without enzymatic removal of the cell wall. We always observed overlap of the mRNA region with CB markers. In CBs indicated by U2B" (Figure 3H–J) and Atcoilin (Figure 3K–M) accumulation of mRNA was frequently observed.

**Figure 4:**
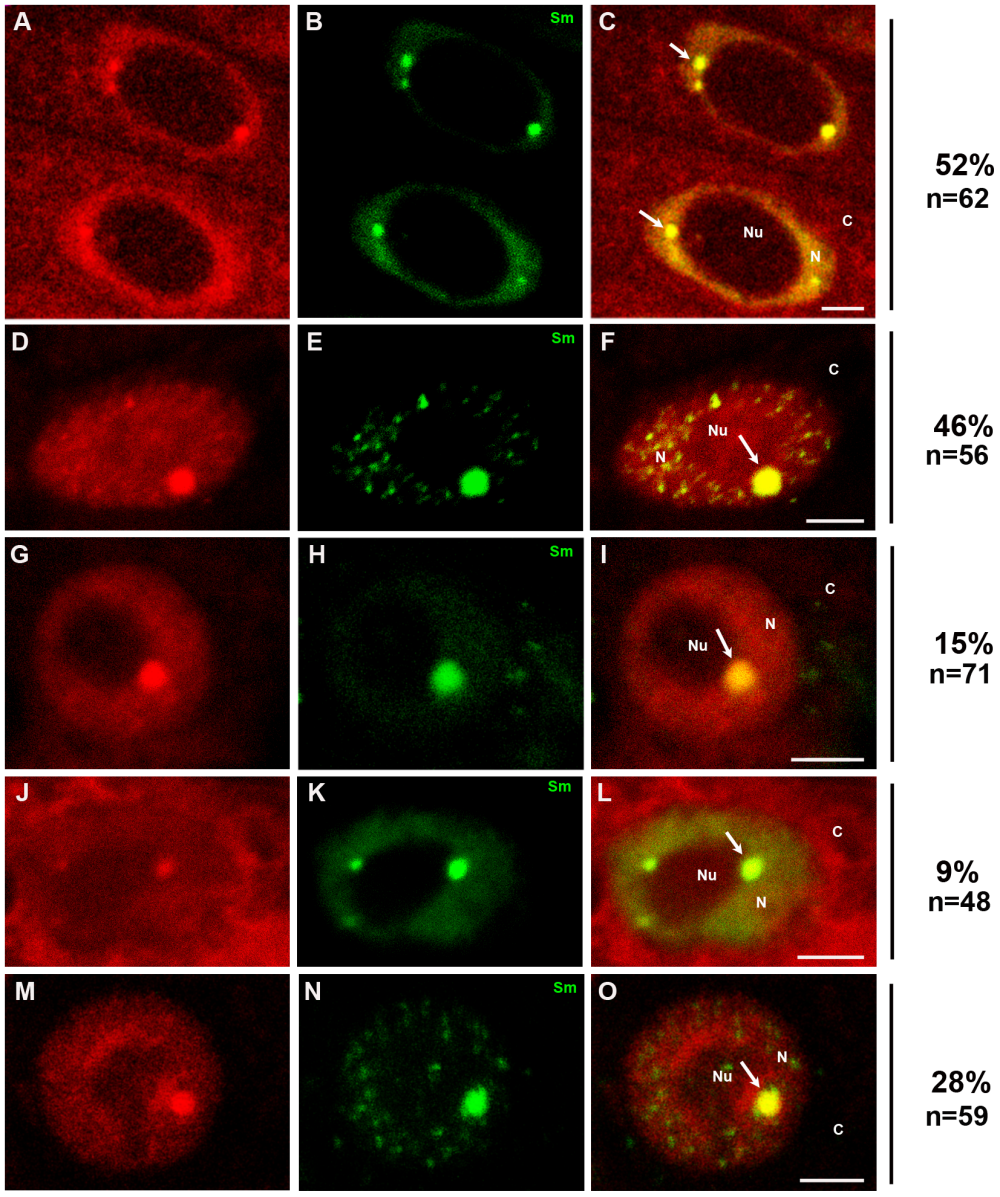
Double labelling of a mixture of four mRNAs (A, C) and distinct genes: cyclin B1 mRNA (D, F), peroxidase mRNA (G, I), cytokinin-specific binding protein mRNA (J, L) and pectin methylesterase mRNA (M, O), with Sm proteins (B, E, H, K, N) in *Lupinus* cells. The arrows indicate colocalisation of mRNA transcripts with CBs. A stronger signal was observed in the cytoplasm than in the nuclei after *in situ* hybridisation with the mixture of probes (A, C) and with a probe against cytokinin-specific binding protein mRNA (J, L). % indicates the percentage of nuclei showing the representative immunolocalisation pattern. Bar, 5 µm. N-nucleus, Nu- nucleolus, C- cytoplasm.

Because poly(A) RNA was localised in plant CBs, we attempted to determine whether some of these RNAs were protein coding. Experiments were performed in *Lupinus luteus* cells, in which the Cajal bodies are large and easily distinguishable after staining with poly(A) RNA and other markers of these structures. The mRNA sequences of four protein-coding lupine genes were chosen for this analysis: a cytokinin-specific binding protein, cyclin B1, pectin methylesterase and peroxidase. Simultaneous localisation of the core spliceosomal Sm proteins and four mRNAs revealed that the CBs, which contain large amounts of Sm proteins, are also the structures that house mRNAs encoding proteins (Figure 4A–C). In addition to localising to the CBs, mRNAs were detected in the cytoplasm and nucleoplasm, except for the nucleolus. To evaluate whether all four of the selected mRNAs occur in CBs, double labelling of Sm proteins and individual lupine mRNAs was performed. As depicted in Figure 4D–O, the cyclin B1 (Figure 4D, F), peroxidase (Figure 4G, I), cytokinin-specific binding protein (Figure 4J, L), and pectin methylesterase (Figure 4M, O) mRNAs colocalise in Cajal bodies with a frequency of 9%–46%. A lack of colocalisation indicates that the signal is present in the cell but is not concentrated in CBs. The highest percentage of colocalisation was observed for the cyclin B1 mRNA (Figure 4D–F), while the lowest was observed for the mRNA of the cytokinin-specific binding protein (Figure 4J–L). The mRNA of the cytokinin-specific binding protein was present at higher levels in the cytoplasm than in the nucleus.

**Figure 5:**
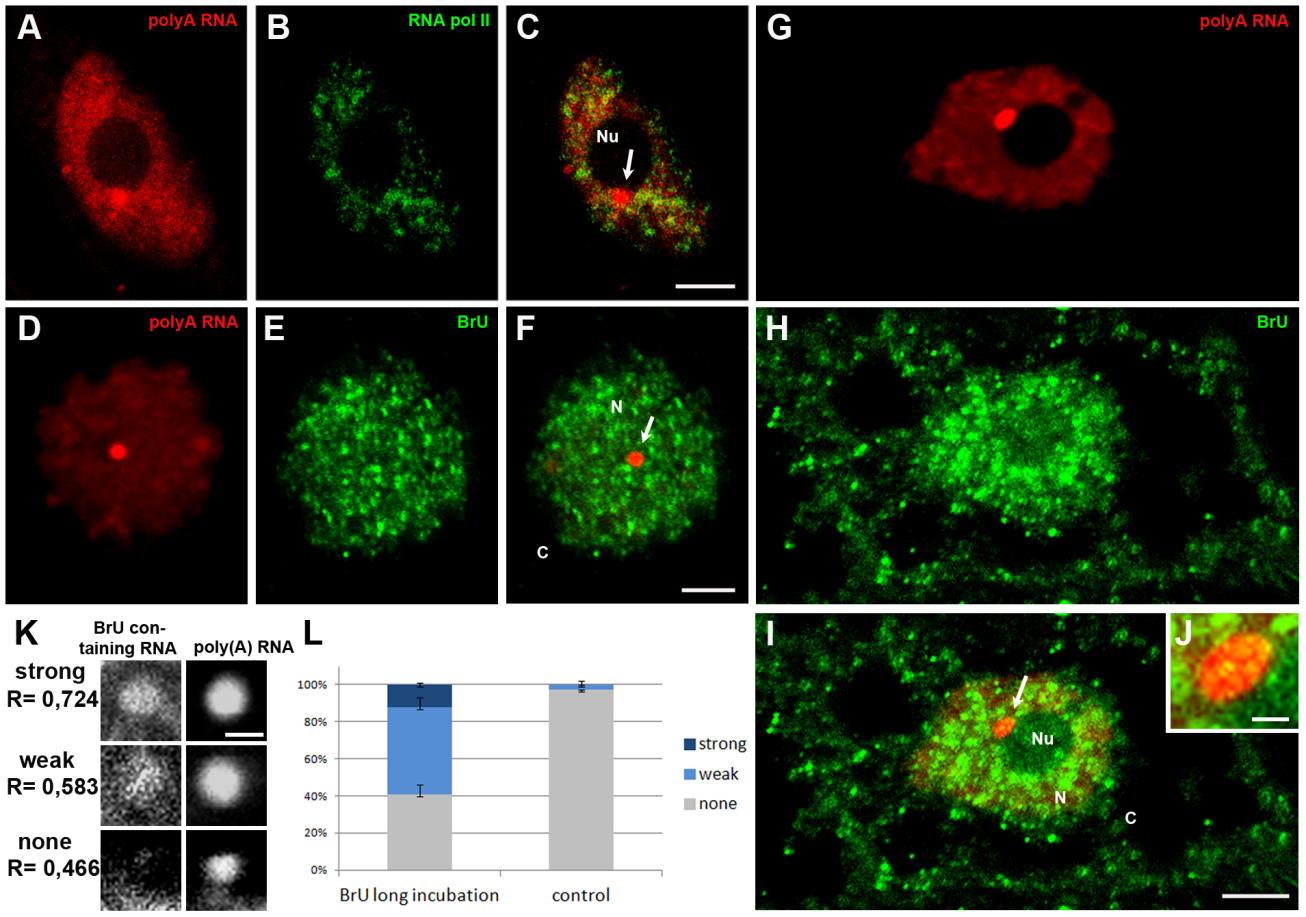
Double labelling of poly(A) RNA (A) and the elongation form of RNA polymerase II (B) in *Lupinu* root cells. Cajal body (arrow) rich in poly(A) RNA near the nucleolus do not colocalise with RNA polymerase II. Double labelling of a newly formed transcript (E, H) and poly(A) RNA (D, G) in lupine cells. If the transcript does not leave the nucleus, no signal occurs in the CB (arrow) (D–F). In cells in which the newly formed RNA is transported to the cytoplasm, a weak signal is observed in the Cajal body (arrow) (G–I). Bar, 5 µm. Fragment of the nucleus from Fig 2I (J). BrU-containing RNA localises at the periphery and in small spots in the middle of CB (J). Bar, 1 µm. The percentage of Cajal bodies rich in poly(A) RNA that colocalise with BrU-containing RNA (K, L). The data for experimental treatments and control were conducted, analyzed and plotted as in Figure 2G–H. A scale bar representing 2 µm is shown. Error bars represent standard error. N- nucleus, Nu- nucleolus, C- cytoplasm.

Next, we tested whether these structures are associated with the transcription process, or with subsequent stages of mRNA maturation. Observations made using an antibody against the elongated form of RNA polymerase II with a phosphorylated serine 2 in the CTD domain revealed clusters of this antigen in the nucleus. The regions of RNA polymerase II localisation did not overlap with the CBs (Figure 5A–C). We also studied whether the new form of RNA accumulates in CBs. In cells in which BrU-containing RNA did not leave the nucleus, the signal formed numerous small clusters. In these cells, no new forms of RNA were observed in Cajal bodies (Figure 5D–F). However, when the treatment time with the analogue of uridine was extended from 2 to 7 hours, BrU incorporated into the RNA was transported to the cytoplasm, and larger brighter spots were observed in the nucleus (Figure 5G–I). The labelled new form of RNA was observed in the centre and at the periphery of Cajal bodies (Figure 5J). Because the observed colocalisation was not unambiguous, we conducted as Pearson correlation as described above. Examples of highly correlated (R> 0,72), weakly correlated (0,72> R> 0,58) and non-correlated (R <0,58) distributions are shown in Figure 5K. Indeed, ∼60% of CBs strongly or weakly colocalised with BrU-containing RNA after a longer treatment time with the analogue (Figure 5L).

**Figure 6:**
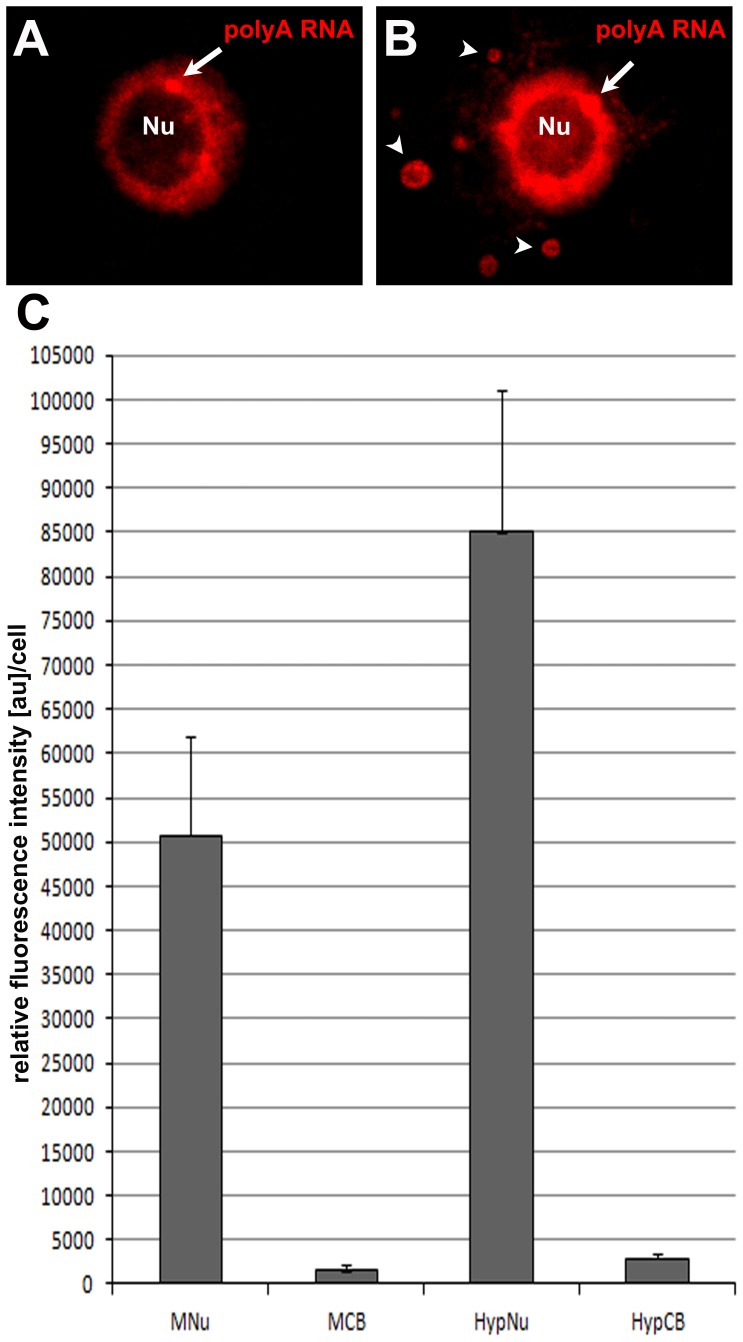
Localisation of poly(A) RNA in control lupine root cells (A) and after submersion to tap water for 3 h (B). Arrows indicate CBs, and arrow heads indicate cytoplasmic granules. Bar, 5 µm. Nu- nucleolus. Quantitative analysis of the fluorescence intensity associated with the localisation of poly(A) RNA. Significant differences in the signal intensity in the nuclei and CBs between control and hypoxia-treated cells (C) MNu- control nucleus, MCB control Cajal bodies, HypNu and HypCB- nucleus and Cajal bodies after hypoxia treatment, respectively (p = 0.05).

Immersion of cut roots or whole seedlings in water leads to hypoxia, which often occurs during flooding [31], [32]. The meristematic cells of lupine showed an increased amount of poly(A) RNA in the nucleus and Cajal-like structures following hypoxia in comparison with the control cells (Figure 6A, B). Simultaneous localisation of the Sm and poly(A) RNA proteins confirmed that large bodies in the root nuclei of seedlings observed after the induction of hypoxia represent CBs (Figure S1I–K). In the cytoplasm, single aggregates were found (Figure 6B). Quantitative measurements showed 1.6- and 1.8-fold increases in the amount of poly(A) RNA in the nucleus and CBs, respectively (Figure 6C). The CBs of hypoxia-induced cells contain approximately 15% more of the nuclear pool of poly(A) RNA compared with the control material.

## Discussion

We have shown that in the cells of plant roots, a considerably larger amount of poly(A) RNA is present in the nucleus than in the cytoplasm (up to more than 80% in lupine). The presence of similarly large amounts of poly(A) RNA in the nucleus has been described previously in many types of animal cells. In comparison with the cytoplasm, larger proportions of both protein-coding mRNA and the total pool of poly(A) RNA are localised in the nucleus in COS-7 cells as well [33]. Many retentive mRNAs have been identified in the nuclei of plant and animal cells to date [34], [35]. Recently, it has also been shown that intron-containing splice variants remain within the nucleus and are not transported to the cytoplasm [36]. Furthermore, it has been demonstrated that non-coding RNAs constitute an important pool of poly(A) RNA in the nucleus, including miRNAs in plants [37]. However, the domains associated with poly(A) RNA metabolism in the nuclei of plant cells are still poorly understood.

The presence of poly(A) RNA in splicing speckles has been reported in animal cells [38], [39], [40], [41]. For the localisation of splicing speckles in plant nuclei, we used the 780-3 antibody against the antigen PANA [42], which is a better marker for these structures than the 3C5 antibody, which recognizes phosphorylated epitope proteins that are components of intechromatin granules [26]. Our results showed that individual round large clusters of poly(A) RNA were not colocalised with clusters of the PANA antigen. This finding indicates that the observed poly(A) RNA-rich bodies are not speckles. Further analyses revealed that these clusters are Cajal bodies. The presence of poly(A) RNA in CBs was observed by our group in both the chromocentric (*L. luteus, A. thaliana* Col-O) and reticular (*A. cepa*) types of nuclei in meristematic and differentiated root cells. To our knowledge this is the first report of the presence of poly(A) RNA in Cajal bodies in somatic plant cells. A similar phenomenon was described by Kołowerzo et al. [24] in the diplotene microsporocytes of larch. However, no mRNA was detected in the CBs of somatic cells of the tapetum. To our knowledge, no information about the presence of poly(A) RNA in Cajal bodies in animals cells has been reported. Recently, the presence of a poly(A)-specific ribonuclease (PARN) was demonstrated in CBs, which is involved in the regulation of mRNA degradation and the enhancement of snoRNA stability [43], [5]. However, no poly(A) RNA was found in these structures. Thus, our results suggest that similar to siRNA biogenesis [4], the presence of poly(A) mRNA in Cajal bodies is a characteristic feature of plant cells.

We have shown that at least a portion of the poly(A) RNAs found in CBs are protein-coding RNAs. However, stages in the metabolism of mRNAs can be carried out in the CB at different intensities or speeds for individual mRNAs encoding proteins, which may be indicated by the frequency of the colocalisation of the four different transcripts of CBs [44], [45], [46], [47]. The presence of protein-coding transcripts (but only those that are synthesised intensively) has also been observed in the CBs of larch meiocytes [48]. At the present stage of this research, the possibility cannot be excluded that the part of poly(A) mRNAs found in CBs are non-coding. However, it is known that these mRNAs are not pri-miRNAs because the presence of pri-163 and 171 with a polyadenylated 3′ end has not been observed in the CBs of *Arabidopsis thaliana* [49], [50]. In contrast, there is a lack of data on the *in situ* localisation of long non-coding RNAs, many of which are polyadenylated [37].

The presence of mRNAs in the CBs of plants, prompts the question of whether Cajal bodies serve as a site of transcription. We have shown that in the CBs of lupine cells, there is a lack of active RNA polymerase II. However, *in situ* run-on transcription assays demonstrated small amounts of transcribed RNA in CBs, but only after a long incubation period when partial transport of a new form of RNA from the nucleus to the cytoplasm was observed. This means that Cajal bodies are not sites of RNA transcription. Similar results have been obtained in animal cells [51], [52]. Our results indicate that the CBs of plant cells are involved in the late stages of the metabolism of poly(A) RNAs, after the termination of transcription, or they are involved in their storage. The splicing some pre-mRNAs occurs post-transcriptionally [53]. The CBs of plants are not a direct site of splicing, as SR proteins are not observed in these nuclear bodies [26].

However, the possibility that CBs participate in the storage or retention of poly(A) RNAs is supported by our observations in hypoxia-treated cells. In cell nuclei after hypoxia, the amount of poly(A) RNA increases by 67%. A similar phenomenon has been observed in *Arabidopsis thaliana* cells after heat and ethanol treatments, which represent two abiotic stresses [54]. In addition, our results demonstrate statistically significant accumulation in Cajal bodies. Following hypoxia, 15% more of the total nuclear pool of poly(A) RNA is found in the CBs compared with control cells. This finding suggests the possibility that Cajal bodies contribute to the retention of mRNA in the cell nucleus. One reason for the retention of RNA in the nucleus is that the process of maturation cannot be completed. Recently, widespread changes in AS in response to developmental cues and stresses have been elucidated. The most frequent of type of AS in plants is intron retention (IR) (∼40%) [55] The IR events in *Arabidopsis* that are predicted targets of nonsense-mediated decay (NMD) escape this mechanism [56] and could result in retention in the nucleus.

In conclusion, our results demonstrate the prevalence of a new Cajal body component, poly(A) RNAs, including protein-coding RNAs, in plant cells. The obtained results do not resolve the role of these structures in the metabolism of poly(A) RNA. However, it has been established that the CBs present in plant roots are not a transcription site but may be involved in the storage or retention of poly(A) RNAs.

## Supporting Information

Figure S1 **Localisation of poly(A) RNA in protoplasts in lupine root cells: meristematic (A), differentiated (B).** Arrows indicate CBs. Double labelling of poly(A) RNA and Sm proteins in: *Allium* (C-E), *Lupinus* (F-H) and hypoxia-treated lupin cells (I-K). Bar, 5 µm. N- nucleus, Nu- nucleolus.
(TIF)
