# Supplementary figures and images for: Poly(A) RNAs Including Coding Proteins RNAs Occur in Plant Cajal Bodies
Source: PLoS One. 2014 Nov 4;9(11):e111780. doi: 10.1371/journal.pone.0111780 (PMC4219776; doi:10.1371/journal.pone.0111780)

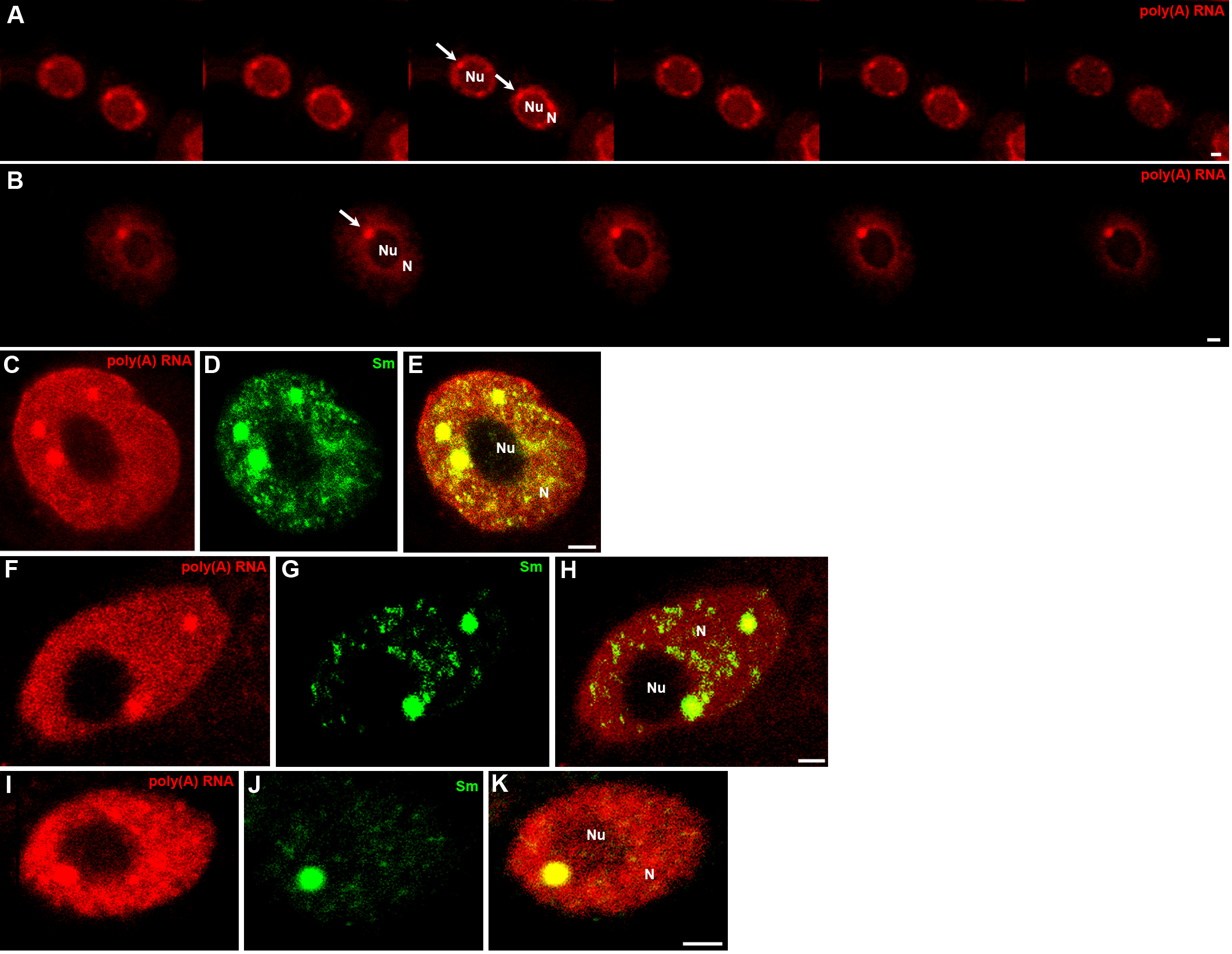

Supplement: Figure S1 — Localisation of poly(A) RNA in protoplasts in lupine root cells: meristematic (A), differentiated (B). Arrows indicate CBs. Double labelling of poly(A) RNA and Sm proteins in: Allium (C-E), Lupinus (F-H) and hypoxia-treated lupin cells (I-K). Bar, 5 µm. N- nucleus, Nu- nucleolus. (TIF) [file pone.0111780.s001.tif]
